# The Developmental and Functional Implications of Age‐Associated B Cells (ABCs) Across Tissue Microenvironments

**DOI:** 10.1111/imr.70162

**Published:** 2026-08-25

**Authors:** Rebecca L. Francis, Jason S. Weinstein

**Affiliations:** ^1^ Center for Immunity and Inflammation Rutgers New Jersey Medical School Newark New Jersey USA

**Keywords:** age‐associated B cells, antibody‐secreting cells, antigen‐presenting B cells, tissue microenvironments

## Abstract

Age‐associated B cells (ABCs) are a dynamic B cell subset whose identity, development, and function are fundamentally shaped by the tissue microenvironments in which they reside. Primarily defined by expression of CD11c and T‐bet, ABCs arise across diverse contexts, including aging, infection, autoimmunity, and cancer, yet exhibit striking heterogeneity that reflects the distinct signals present in each tissue niche. While the core generative cues of TLR7/9 activation, IFN‐γ, IL‐21, and BCR stimulation are common, the local tissue environment dictates how these signals are integrated and which functional properties are ultimately expressed. In infectious environments, altered splenic structure, peripheral tissue inflammation, and organ‐specific cytokine gradients influence context‐dependent ABC phenotypes with unique localization and effector characteristics. In autoimmune diseases, target organ microenvironments provide the TLR ligands, cytokines, and cellular interactions that sustain local ABC expansion and pathogenic activity. Similarly, in the tumor microenvironment, ABCs are shaped by tertiary lymphoid structures and chronic inflammatory signals that can shift their function between pro‐inflammatory and immunosuppressive states. This review explores how tissue microenvironments influence ABC biology in infections, autoimmunity, and cancer, highlighting critical implications for understanding disease mechanisms and developing targeted therapeutic strategies.

## 1 Introduction

The dynamic signals in our tissue microenvironments play a substantial role in generating the heterogeneity we see across immune cell subsets. During an infectious insult or disease state, our microenvironment reacts to create a milieu that, ideally, counteracts the perturbation and restores homeostasis. These environmental cues prime cells to be receptive to signals that shape a cell's identity, localization, and function. Generating inappropriate cellular characteristics can lead to responses that are ineffective at clearing pathogens or promote the development of pathology. Thus, understanding the cues that drive optimal cell subsets for a particular situation, along with their local and systemic effects, is important for developing therapies that can fine‐tune the development of an efficient response.

**FIGURE 1:**
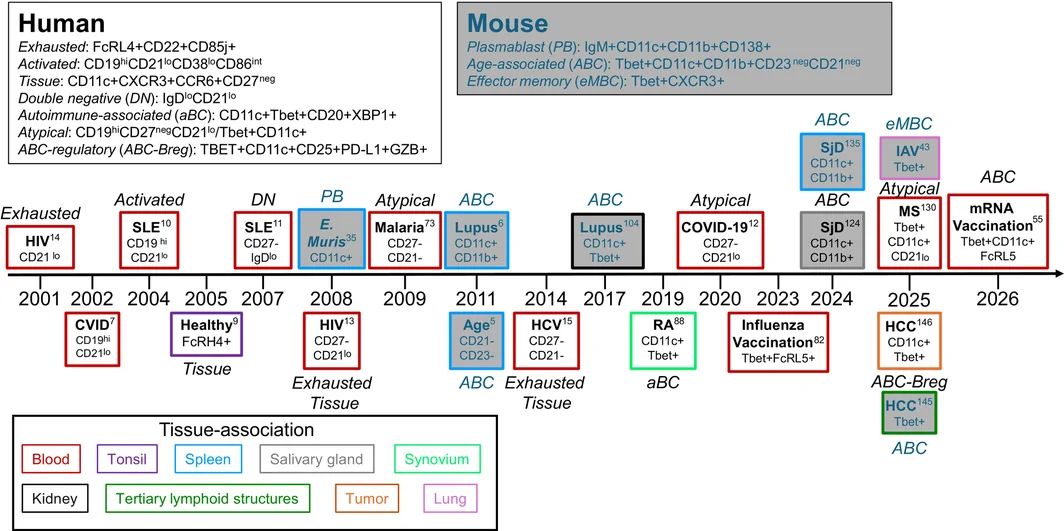
Timeline of ABC discovery in mouse and human. Detailed are ABC's disease contexts, defining markers, tissue‐association (indicated by outline color), and phenotypic descriptions in mice (gray fill) and humans (white fill). Expanded boxes for mice and humans detail specific, defining markers. Numbers represent the reference article. CVID, common variable immunodeficiency; HCC, hepatocellular carcinoma; HCV, hepatitis C virus; HIV, human immunodeficiency virus; IAV, influenza A virus; MS, multiple sclerosis; RA, rheumatoid arthritis; SjD, Sjogren's disease; SLE, systemic lupus erythematosus.

B cells are generally categorized based on their functions or anatomical locations. In general, their terminal effector phenotypes are antibody‐secreting cells (ASCs), which arise from different differentiation pathways, such as germinal center (GC) or extrafollicular (EF) [1, 2]. Prior to development into ASCs, they can serve intermediate roles as memory B cells (MBCs), antigen presenting cells (APC), or cytokine secreting cells [3]. Unsurprisingly, these developmental or functional pathways depend on the history of signals and environments that the B cells have experienced. A recently identified alternative differentiation or functional stage of B cells, the age‐associated B cell (ABC) [4], highlights this complexity of B cell differentiation. While the consensus is that these cells are generated during Type 1 immune conditions, there are often conflicting characteristics or functional attributes for this B cell subset (Figure 1). This could be due to various defining markers used across studies, or the diverse contexts in which they are found that promote different defining features of this subtype.

Studies have used various descriptions and markers for identifying ABCs. The age‐associated designation comes from their accumulation in aged mice as B cells that uniquely express the integrin CD11c and the transcription factor T‐bet, or as being CD23^−^CD21^−^ [5, 6]. However, a similar subset was described in a variety of contexts in humans prior, including in common variable immunodeficiency (CVID), systemic lupus erythematosus (SLE) patients, in tonsils of healthy individuals and during infections [7, 8, 9, 10, 11, 12]. These contexts describe them as atypical MBCs or double negative (DN) B cells, as they are IgD^lo^ cells that lack the conventional memory marker CD27 and are commonly identified as CD21^lo^, features that distinguish them from classical MBCs. DN B cells are further subdivided into 4 subsets based on their expression of CD11c and T‐bet, with DN2 being the ABC mouse equivalent by expressing CD11c and T‐bet. In addition, early studies of chronic infections characterized similar B cells with increased expression of markers known to dampen B cell receptor (BCR) signaling, such as FcRL4 and FcRL5, thus classifying them as exhausted cells in human immunodeficiency virus (HIV), hepatitis C virus, and malaria infections [8, 9, 13, 14, 15]. Thus, it was hypothesized that the chronic exposure to antigen in these diverse environments promotes this B cell subtype.

Given these distinct marker classifications, the diverse environments in which they are found, and the differing cells used for comparison, it is no wonder that these cells constitute a very heterogeneous population that can appear to have characteristics of memory, activated naïve, antibody‐secreting, tissue‐associated, and exhausted B cells. Much of this heterogeneity remains to be fully elucidated, limiting our understanding of their unique contributions to the humoral response. Despite these differences, there are some commonalities in their generative signals that promote specific features in a given context. Here, we will discuss the common generative signals of ABCs and how their specific disease context and microenvironment support developmental and functional attributes that may explain some of the heterogeneity of this subset.

## 2 Drivers of ABCs

Conventional B cell activation begins with BCR ligation to antigen. The nature of the antigen can have varying effects depending on the B cell binding to it. For antigens that contain highly repetitive epitopes, crosslinking of multiple BCRs can induce T cell independent activation for various subsets, often leading to a short proliferative burst and direct differentiation into low‐affinity short‐lived IgM ASCs. Alternatively, the most effective B cell responses are generated by a T‐dependent pathway. T cell help leads to higher specificity and functionality of the BCR, as well as promoting longevity. There is tremendous heterogeneity in the nature of these responses, depending on the interacting partners and environmental signals. While antigen specificity is a unique feature of B cell activation, other factors, such as cytokines, toll‐like receptors (TLRs), and T cell interactions, play important roles in dictating the B cell's fate, including isotype switching, ASC differentiation, and affinity maturation, respectively.

ABCs are considered an antigen experienced population, whose BCRs are often found to be poly‐ or auto‐reactive, but also exhibit high specificity for infecting agents. While BCR ligation has been shown to be important for initial differentiation, patients with BCR‐signaling deficiency have reduced CD21^lo^ B cells [16], and ABC proliferation is refractory to BCR ligation alone [5]. This may be due to the high levels of BCR inhibitory receptors, such as FcRL4/5 or CD22, on their surface. This dampening of BCR signals may make other stimuli more important for further ABC differentiation and function. TLR signaling is essential for the generation of ABCs, specifically through TLR7 and TLR9, as mice that lack these receptors or humans with loss‐of‐function mutations have reduced generation of ABCs [5, 6, 17]. How these TLR ligands are delivered to promote activation, whether by BCR‐ or other receptor‐mediated endocytosis, or internal ligands, is not completely understood. Additionally, TLR4/3/2 deficient mice showed no effect on ABC accumulation, suggesting a specificity for TLR7 and TLR9. However, a deficiency in MyD88, the adaptor protein for most TLRs, also resulted in reduced ABC accumulation [5]. This could suggest other interacting partners or pathways downstream of TLR7/9 signaling that are independent from canonical signaling, as shown for TLR9 [18]. Although there is consensus on the importance of TLR signaling in this subset, there remains a lack of understanding as to why they are specifically sensitive, given that TLR signaling is important for activation/differentiation of B cells in general.

**FIGURE 2:**
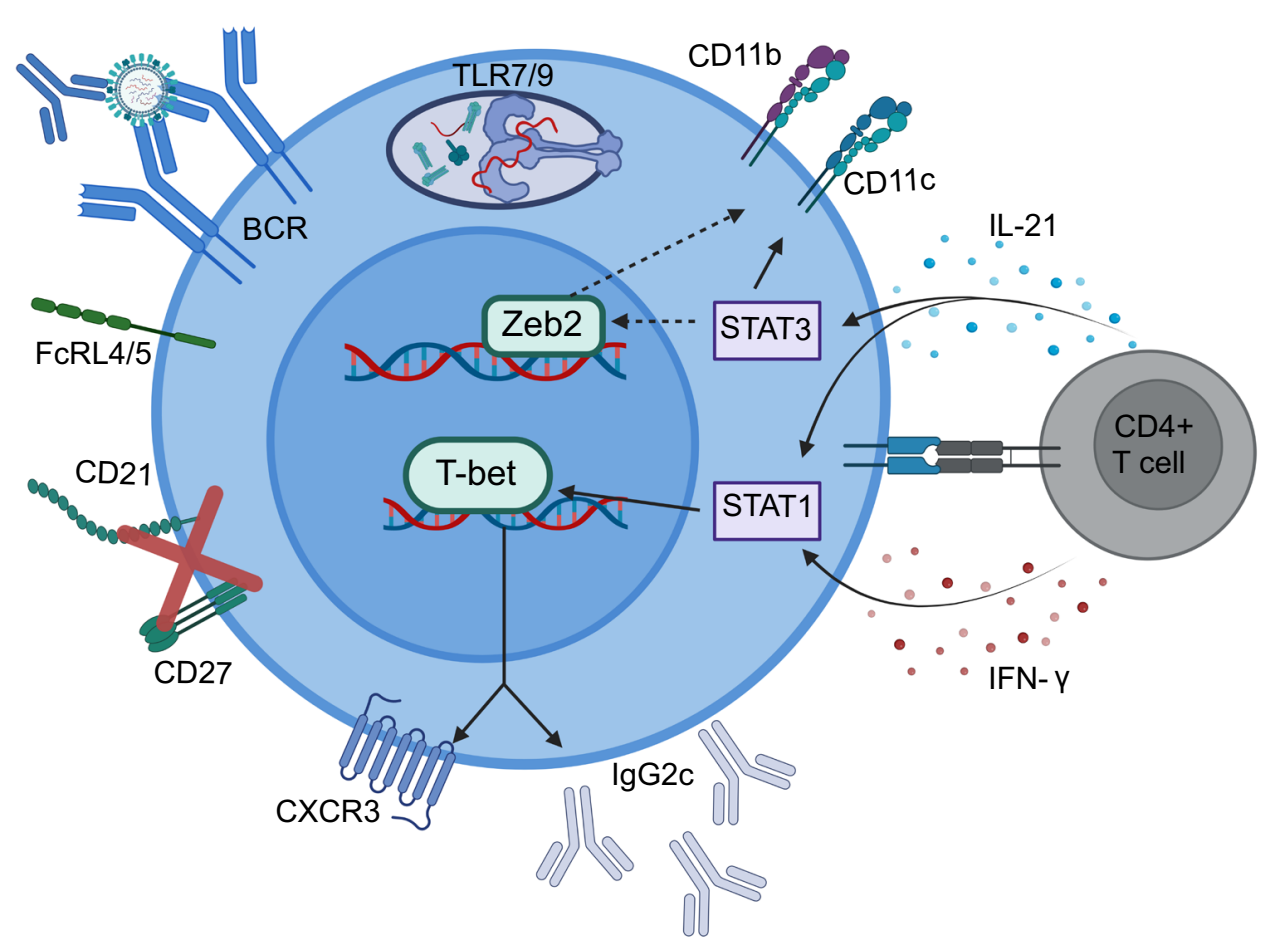
Common generative signals and features of ABCs across disease contexts. ABCs express a distinct profile of inhibitory receptors, integrins, and transcription factors, while lacking conventional B cell markers such as CD21 and CD27. BCR and TLR signaling are initial triggers that promote activation and upregulation of inhibitory receptors such as FcRL4/5. CD4^+^ T cells solidify ABC identity by secreting IL‐21 and IFN‐γ. IFN‐γ signals via STAT1 to induce T‐bet expression, which drives CXCR3 expression and class‐switch recombination to IgG2a/c. While IL‐21 can also activate this STAT1 pathway, it primarily signals through STAT3 to upregulate CD11c expression. Zeb2 is also suggested to be induced by IL‐21 and promote CD11c expression, though the exact signaling pathway is not fully understood.

Cytokines are necessary for promoting appropriate B cell differentiation. IFN‐γ is a well‐known driver of T‐bet expression in B cells and has been demonstrated to be necessary for ABC differentiation [19, 20, 21]. It has been shown that signaling through IFN‐γR promotes STAT1 activation and this pathway increases T‐bet expression under a variety of conditions. In contrast, deficiency in Type 1 interferons (IFN‐Is) was not shown to affect ABC generation. IL‐21 is another cytokine that is necessary for ABC generation. IL‐21 signals primarily through STAT3, but also through STAT1 and STAT5 [22]. In ABCs, these signals are necessary for both CD11c and T‐bet expression, with STAT3 signaling promoting CD11c and STAT1 promoting T‐bet expression [20, 23]. While T‐bet was reduced in mice with B cells lacking either IFN‐γR or IL‐21R, CD11c was reduced only in IL‐21R‐deficient mice [23]. This supports the idea that, although many signals promote T‐bet, CD11c expression is more restrictive. Regardless, CD11c and T‐bet expression on ABCs is not absolute, and cells similar to ABCs can be found lacking either marker [24, 25, 26, 27, 28]. Also, IL‐4 can inhibit IL‐21‐driven T‐bet expression, whereas IFN‐γ‐driven expression is not affected [20]. This supports an uncoupling of signals that drive T‐bet and CD11c, which likely depend on the environmental contexts. As most studies support dual stimulation by IFN‐γ and IL‐21 for optimal ABC generation, the most likely sources of these cytokines have been established. While IFN‐γ can be produced by a variety of cells, IL‐21 is primarily produced by CD4^+^ T cells, specifically Th1 and Tfh cells. While some studies show T‐independent generation of ABCs, most support that they are T‐dependent [16, 28, 29, 30]. These studies show their proximity to Tfh during viral infection, rather than Th1, and that several Tfh knockout models‐ Icos^−/−^, Sh2d1a^−/−^, Bcl6^fl/fl^CD4^cre^‐do not generate ABCs [28, 29]. Thus, the prevailing model for ABC generation is TLR7 or TLR9 stimulation accompanied by Tfh cell help that delivers IFN‐γ and IL‐21 (Figure 2).

The microenvironments in which B cells develop and differentiate confer distinct characteristics. Historically, assessing these routes of development has been restricted to secondary lymphoid tissues either within the GC or outside of the follicles in the EF regions; however, routes such as interfollicular or peripheral tissue development are now recognized as additional differentiation environments [31, 32]. ABCs were originally characterized as being GC‐derived because they have higher mutational burdens than naïve cells [4]. As the role of non‐GC‐derived somatic mutations and isotype switching has now been appreciated [33, 34], GC‐fate‐mapping studies have supported that only about 15% of ABCs actually have a GC origin [29]. Several localization studies have identified them in splenic red pulp near the marginal zone or at the T‐B border, classifying them as part of the extrafollicular response [29, 35, 36]. It is unclear whether GC‐experienced ABCs are generated in the GC or whether a GC‐derived precursor cell can further differentiate into ABCs. If they follow the typical attributes of GC vs. EF B cells, GC‐derived cells may have higher affinity for antigen or be long‐lived, playing important roles during secondary insults; whereas, EF‐derived cells may support a rapid effector response. The development of ABCs in the GC or EF could be influenced by the environmental milieu, as inflammatory conditions, such as high levels of IL‐12 or IFN‐γ, can promote EF activation at the expense of GC development [37]. ABCs can also be found in peripheral tissues, and this accumulation is not necessarily dependent on their generation in lymphoid tissues [23, 38]. Thus, ABC localization and developmental niche likely impart unique signals that support functional or survival characteristics.

ABC identity is shaped by a distinct transcriptional network, primarily driven by T‐bet and Zeb2. T‐bet expression in B cells is a well‐known regulator of class switch recombination, promoting IgG2a/c in mice and IgG1 in humans [39, 40, 41]. Apart from this, additional functions or the lineage determination of T‐bet in B cells were not well studied, particularly regarding persistent expression. However, in the last several years, additional roles of T‐bet in B cells have been discovered, including promoting ASC differentiation by inhibiting the IFN‐γ‐induced inflammatory gene program that restricts differentiation and promoting various migratory properties that depend on the level of T‐bet expression [27, 42, 43]. While T‐bet levels are elevated in ABCs, several studies have demonstrated that B cells deficient in T‐bet can still generate ABCs, with the major defect being the loss of isotype switching to IgG2c [24, 44]. This suggests that T‐bet is not necessary for their generation or identity, but rather for survival, differentiation, and fine‐tuning functional attributes. Zeb2 is another transcription factor that is highly expressed in ABCs, and its reduction in mice or humans results in decreased ABC numbers. This decrease is due to deficiencies in promoting ABC identity, unlike T‐bet, which is also needed for cell survival [45, 46, 47, 48]. Zeb2's role in ABCs is not fully understood, but it has been shown to regulate CD11c expression [49]. Zeb2 plays a role in promoting conventional dendritic cell (cDC2) differentiation, which is required for presentation to CD4+ T cells [50]. Whether Zeb2 could exert similar effects in B cells is unknown [51], but it would suggest a balance between the influences of T‐bet or Zeb2 on ABC function, supporting survival/ASC differentiation or identity/T cell interactions, respectively.

Taken together, the most universal signals for ABC generation are T cell help, IL‐21 and IFN‐γ, BCR stimulation, and TLR signaling (Figure 2). The variations in marker expression, especially ABC's being identified with both positive and negative T‐bet and CD11c expression, suggest that there is a decoupling or independent regulation of their most universal markers. It is likely that these represent different stages of differentiation based on the environmental signals they are responding to, or they could represent an entirely different subset. However, various combinations of the generative signals can promote the ABC phenotype to varying degrees. IFN‐γ stimulation alone can upregulate T‐bet expression, but further increases are observed upon the addition of anti‐IgM, TLR ligands, or anti‐CD40L [16, 20]. In vitro stimulation with a combination of BCR, IFN‐γ, CD40, and IL‐21 is required to generate the CD21^lo^ ABC phenotype, but similar results are observed when substituting T cell help for TLR signaling [16]. In addition, the timing of the signals can affect the extent of ABC generation, with IFN‐γ or TLR signaling being needed earlier and T cell help later during activation [20]. Early innate signals promote rapid proliferation and differentiation of B cells, which otherwise undergo apoptosis unless given T cell help [52]. This first wave of signals may allow B cells to be more receptive to T‐cell signals, thereby supporting survival and further ABC differentiation. While strong TLR signaling in vitro may serve as a substitute for T cell help in ABC generation, patients with various inborn errors of immunity suggest that defects in T cell help result in greater reductions of CD21^lo^ cells than those of TLR deficiencies [16]. The low number of patients with these deficiencies makes it difficult to draw strong conclusions, but it suggests a fundamental difference between in vitro and in vivo ABC generation. Thus, while T cell help is necessary in most cases, this also suggests that environments enriched in TLR ligands may be able to generate ABCs independently of T cell help. These data suggest that multiple signals could be used to generate ABCs with varying degrees of efficiency.

A key feature of receiving these generative signals is the tissue that allows such interactions. ABCs are most expanded in the spleen during many insults [29, 35], and are rarely found in lymph nodes (LNs) [5, 27, 43, 53]. However, follicular B cells taken from LNs can be stimulated in vitro to generate ABCs. Because the BCR, TLR, and T cell help signals that generate ABCs are present in all lymphoid organs, this suggests that additional splenic stimuli selectively promote ABC generation in the spleen. ABCs are also found in peripheral tissues, and studies suggest that this phenotype can be generated in inflamed organs independently of their splenic counterparts [23, 38]. Whether this is truly *de novo* B cell generation or further differentiation from other activated precursors remains unclear. The functions of ABCs are still under investigation, with a wide array of roles being attributed to them, including cytokine production, antibody‐secreting cell precursors, antigen presenting cells, and marginal zone‐like memory cells [4, 29, 48, 53, 54]. Furthermore, while ABCs can perform these functions, it is unclear what they do in situ and how they differ from other B cell subsets that perform similar functions. Some studies show enhanced ASC transcriptomes or antigen presentation capabilities compared to other B cell subsets, while others do not [36, 42, 43, 53, 54, 55]. These functions are not mutually exclusive and likely depend on the environmental cues that would necessitate each phenotype. Therefore, understanding the microenvironments of the diseases in which ABCs are found can help shed light on the variety of stimuli these cells receive that contribute to their heterogeneity, and the downstream functional consequences of these signals that influence their role in disease. It is clear that they are important immune players in aging, acute and chronic infections, autoimmunity, and cancer.

## 3 Infection

### 3.1 Infections With Lymphoid Organ Disorganization

**FIGURE 3:**
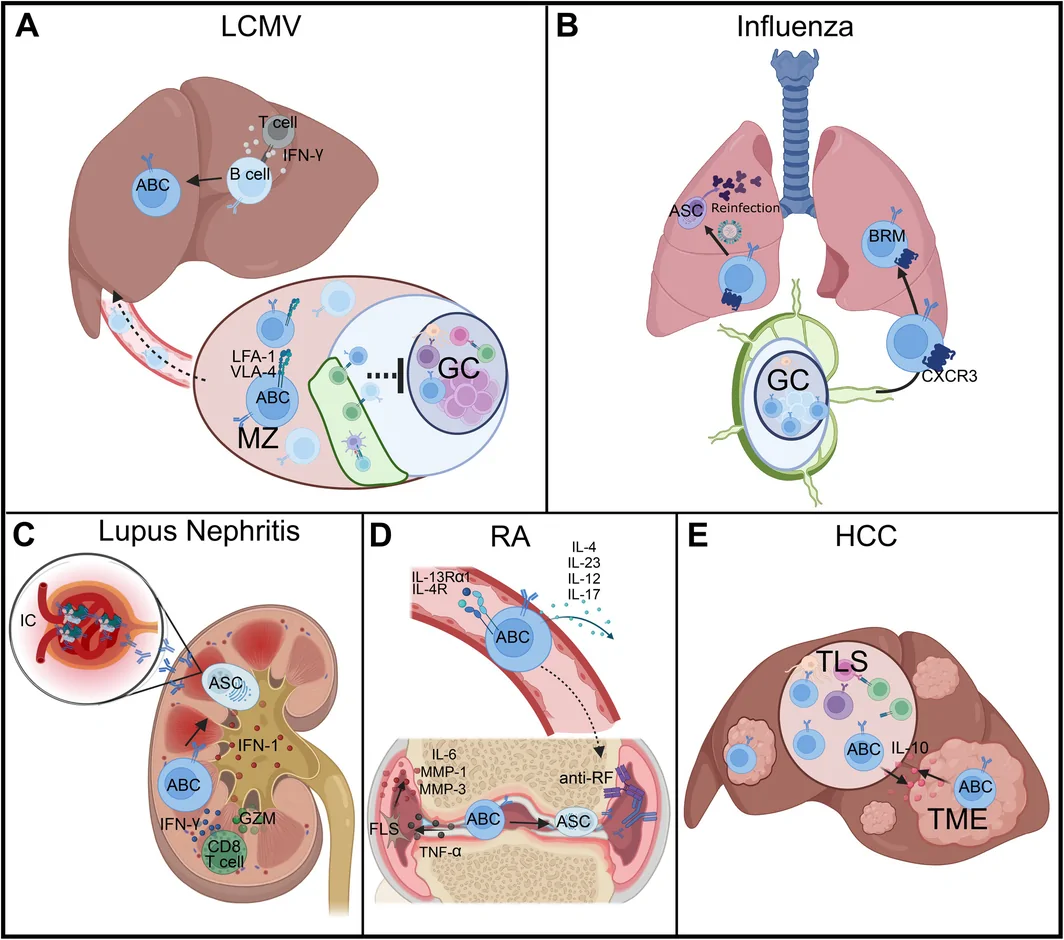
Tissue‐associated characteristics of ABCs. (A) LCMV infection disrupts lymphoid architecture that transiently inhibits GC responses but promotes ABC expansion primarily through EF development. Long‐term retention of ABCs in MZ is promoted by integrins LFA‐1 and VLA‐4. Liver generation is also seen, but the precursors or signals that direct these responses are less understood. (B) After influenza infection, T‐bet^+^CXCR3^+^ B cells arise from GC‐precursors in LN to become BRMs that differentiate to highly specific ASCs after reinfection. (C) Lupus nephritis is a major complication during SLE pathogenesis. ABCs develop locally in the kidney milieu in response to this highly inflammatory environment, driven by local IFN‐I and IFN‐γ and GZM from CD8^+^ T cells. Differentiation of ASCs promotes immune complex deposition in the glomerulus, thereby furthering kidney damage. (D) ABCs are found in the blood and synovium of patients with RA, where they differentiate into ASCs to contribute to anti‐RF pathology. They secrete TNF‐α, which activates FLSs, which in turn secrete cytokines and enzymes to create an inflammatory loop in joints. (E) ABCs are found in TME and TLS during cancer progression. They can promote anti‐tumor immunity by supporting TLS formation, but also secrete IL‐10, leading to immunosuppression. ASC, antibody‐secreting cell; BRM, resident memory B cell; FLS, Fibroblast‐like synoviocytes; GC, germinal center; GZM, granzyme; HCC, hepatocellular carcinoma; IC, immune complex; LCMV, lymphocytic choriomeningitis virus; MZ, marginal zone; RA, rheumatoid arthritis; RF, rheumatoid factor; TLS, tertiary lymphoid structure; TME, tumor microenvironment.

ABCs are found in a variety of acute and chronic infections. One commonality of these environments, where they are most expanded, is infections that exhibit a disruption of the normal lymphoid architecture, particularly in the spleen. These infectious agents—like *Salmonella*, *Ehrlichiae*, and lymphocytic choriomeningitis virus (LCMV)—all cause altered splenic architecture that leads to inhibition of the GC response [56, 57, 58]. It is not fully understood what is causing the inhibition of the GC pathway, and it is likely due to mechanisms that may be pathogen‐specific. One such cause could be the destruction of cells located in the marginal zone (MZ) or red pulp of the spleen. During LCMV infection, virally infected cells include macrophages, dendritic cells, and sometimes lymphocytes that reside in these locations [56, 59]. It has been shown that CD8^+^ T cells are responsible for killing LCMV‐infected antigen‐presenting cells and macrophages in the marginal zone, leading to architectural disruption 6–8 days after infection and transient immune suppression [56, 60]. This cellular destruction not only depletes the cells responsible for activating other immune cells but also causes zonal mixing, which can result in a lack of cues that support appropriate interactions. During *E. muris* infection, TNF‐α production disrupts CXCL13 gradients, thereby inhibiting the GC response and leading to disorganized environments [61]. Despite this, non‐GC responses are still generated, highlighting the B cell populations that can arise in the absence of lymphatic organization. These cells were originally classified as non‐specific IgM cells that primarily differentiate into short‐lived plasmablasts [35]. However, studies have demonstrated they are not non‐specific but rather low‐affinity, as during *Salmonella enterica* serovar Typhimurium (STm) infection in BCR‐restricted mice, B cell effectors are reduced [62]. Additionally, it is established that somatic hypermutation and class‐switch recombination occur not only in GC responses but also in EF pathways. The low‐affinity cells activated in the EF pathway undergo affinity maturation but at a much slower rate than GC B cells, as the GC is optimized for this process. Despite splenic architecture disruption, effective B cell responses occur that are skewed toward generating fast‐acting cells needed to control the ongoing, highly inflammatory or high pathogen burden environment, rather than the slower response of generating high affinity memory cells in the GC [37]. The expansion of CD11c^+^T‐bet^+^ B cells in these responses (Figure 3A) suggests that they are particularly promoted and optimized for such environments.

Despite some commonalities, ABCs exhibit unique characteristics between these infections. In *E. muris* infection, most CD11c^+^ cells are IgM+ and produce little IgG despite expressing T‐bet [25, 35]. This contrasts with LCMV infection, in which about 50% of CD11c + T‐bet^+^ B cells were IgG2c/b^+^ and exhibited an isotype profile similar to that of GC B cells [29]. This could be due to a couple of reasons: one being the differing requirements of T cells, and another being the ability for GC development. The CD11c^+^ plasmablasts in *E. muris* infection could be generated in a T‐independent manner, as B cells lacking MHC‐II were still generated [35]. Further phenotyping of these cells revealed that they expressed high levels of MHC‐II and costimulatory molecules, such as CD40, CD80/86, suggesting that they can interact with T cells but are not strictly required to do so. However, T‐bet^+^CD11c^+^ B cells required T cell help, indicating distinct requirements for effector generation [28]. T‐bet^+^CD11c^+^ B cells in LCMV infection required Tfh cells for their generation, as several Tfh knock‐out mouse models failed to generate ABC [29]. The T cell cytokines IL‐21 and IFN‐γ drive the ABC phenotype by promoting CD11c expression through IL‐21 STAT3 signaling, and T‐bet through IL‐21 and IFN‐γ STAT1 signaling, as mice lacking these receptors showed reduced marker expression and ABC generation [20, 23]. While multiple T cell subsets can secrete these cytokines, it was specifically demonstrated that Tfh cells, and not Th1 cells, were required for ABC generation, as both adoptive transfer and proximity experiments showed [29]. Interestingly, STm infection has not been demonstrated to generate canonical ABCs, which may be due to a high IL‐12/IFN‐γ environment that represses Tfh cell generation and B cell T‐bet expression [37, 57]. During these infections, the GC is transiently shut down, but GC‐like aggregates can be generated, and GC responses are eventually restored. Using S1PR2^CreERT2^ Rosa26^Lox‐Stop‐Lox‐tdTomato^ GC reporter mice, LCMV and influenza infection promoted 10%–20% of ABCs to be derived from the GC by day 10 post infection [29]. While the timing or effects of the GC on ABC development are unknown, it is possible that the higher mutational frequencies, switched isotypes, and long‐lived properties seen in T‐bet^+^ B cells could be heavily influenced by the subset that develops in the GC environment.

Although ABC generation occurs primarily in the spleen and rarely in the LN, peripheral tissue responses are also observed. Analysis of B cell clones in the spleen and liver during *E. muris* infection has demonstrated the connectivity of clones between the two sites, but also highlights clones specific to one site or the other [38]. Mice infected with LCMV that are unable to establish proper splenic environments, either from lymphotoxin‐α^−/−^ mice or splenectomy, do not generate ABCs in the spleen, but still generate similar numbers in the liver, suggesting potential de novo B cell activation and development in peripheral tissues [23]. An alternative mechanism is an unknown precursor cell that migrates to the liver and receives local signals that promote ABC phenotypes (Figure 3A). Indeed, blocking lymphocyte migration with FTY720 early in LCMV infection prevented the generation of ABCs in the liver, suggesting a precursor cell is required [23]. As *E. muris* and LCMV can infect the liver, it is not surprising that the liver is equipped with IFN‐γ and TLR signaling pathways to promote differentiation. However, if local T cell signals are required, or which subset is needed to generate ABCs locally, is not known. As transcriptional profiles of B cells found in the spleen and liver differ, it is possible that the signals required for ABCs to develop in the spleen would not be needed in peripheral tissues [38]. Therefore, the exact tissue milieu likely provides unique signals that ensure survival in that environment and potential differentiation. Whether ABCs persist as tissue resident cells or have tissue‐specific functions remains unclear, but B cells in non‐lymphoid tissues can perform a wide range of effector functions, such as cytokine production, antigen presentation, and ASC differentiation [63].

ABC generation in EF responses is supported by their enrichment in regions of the spleen, in or adjacent to the marginal zone [29, 35]. This localization appears to be temporal, as on day 12 post LCMV infection, T‐bet^+^CD11c^+^ B cells are located close to the white/red pulp border, but only a few are found within the MZ, as shown by imaging and systemic intravenous (i.v) labeling to measure blood‐accessible regions [29]. However, after infection resolved on day 30, 70% of T‐bet^+^CD11c^+^ B cells were i.v. labeled, compared with 40% of other memory B cell populations. It is common for ASCs and memory B cells to relocate to the red pulp regions following infection resolution, thereby supporting long‐term splenic survival [64, 65]. Here, they can protect against immediate reinfection by secreting antibodies into the blood and are optimally located to respond to systemic antigens. While T‐bet^+^ B cells make up only about 5% of the total i.v. labeled B cells at infection resolution, their preference for localizing to these regions could suggest an enhanced ability to reside in these locations. Indeed, several studies have found they have high levels of integrin expression needed for adherence in the MZ, including CD11c, CD11b, VLA‐4, and LFA‐1 [29, 66]. As such, anti‐VLA‐4 and anti‐LFA‐1 blocking antibodies can reduce localization to the MZ with a subsequent increase in blood [29]. Once reorganization of the marginal zone has occurred post‐infection, T‐bet^+^ B cells have been shown to primarily occupy this niche rather than naïve MZ B cells [38]. MZ B cells do not express T‐bet, leaving uncertainty about why the niche is populated with ABCs. It is possible that ABCs can repopulate more quickly or outcompete other B cells. Moreover, it is tempting to speculate that the ABCs in the red pulp might have functions similar to those of MZ B cells, given their similar locations. These functions include blood sampling of systemic antigens that can lead to direct ASC differentiation, antigen shuttling to DCs, or direct antigen presentation to T cells. MZ B cells express high levels of CD21, which can bind pathogen antigens via complement components, thereby shuttling them to DCs to promote T cell activation. ABCs have low CD21 expression, suggesting that this mechanism is unlikely. However, high antigen presentation markers, increased affinity maturation, and high levels of chemokine receptors could suggest a more specific and direct presentation to T cells.

### 3.2 Chronic Infections

Chronic infectious environments lead to altered immune responses due to viral‐induced changes, such as immune evasion mechanisms, excessive inflammatory mediators, and persistent pathogens, which together result in defective protective responses [67]. In settings such as HIV or malaria infections, ABCs are described as atypical B cells, and they can expand to comprise up to 20%–50% of B cells in peripheral blood [13, 68]. These atypical B cells are typically identified as CD27^−^CD21^lo^ cells that express high levels of T‐bet and inhibitory markers such as FcRL4, FcRL5, and CD85j [13, 69]. These inhibitory markers have been a key defining feature of atypical B cells, as high expression of these receptors can explain their poor response to BCR stimulation. Early studies in HIV patients had likened these characteristics to exhaustive phenotypes seen in T cells [13]. However, regarding T cell exhaustion, these attributes do not render cells inert but rather confer altered effector properties. Whether these properties are protective or pathogenic may depend heavily on the disease context. Several studies have investigated the ABC subset in protective acute or chronic pathogenic contexts to understand their dysregulation under prolonged inflammatory settings. Comparing the transcriptomes of ABCs taken from HIV, malaria, autoimmune, or healthy donors has revealed that ABC gene signatures are similar across disease contexts [69, 70]. Additionally, comparing the chromatin accessibility of T‐bet^+^ cells taken from acute versus chronic LCMV infection revealed that only 6 genes were differentially accessible [71]. These studies highlight that ABCs do not seem to be dysregulated in chronic settings, but rather the environment is optimal for supporting their generation.

In the context of chronic infections, ABCs form a distinct lineage driven by their conserved signaling pathways. Trajectory analysis of B cell population in the blood of malaria‐exposed individuals shows that classical and atypical MBCs are derived from similar naïve precursors. They diverge into two branches, with the ABC branch having a heightened IFN‐γ gene signature [70]. As in previous studies, Zeb2 was highly correlated with the ABC gene signature and CD11c expression in malaria infection. Knocking down Zeb2 or IL‐21R reduced ABC formation, suggesting IL‐21 signaling promotes Zeb2‐mediated upregulation of CD11c expression [51]. The absence of Zeb2 also reduced Tfh cells, suggesting that ABCs either help maintain or activate T cells to differentiate into the Tfh cell population, supporting the idea that reciprocal signals are needed for each cell type. This interaction is likely to occur outside of GCs, as T‐bet^+^ B cells were not localized in GCs of the LN in HIV patients [72]. Atypical B cells have similar isotypes and mutational frequencies as other MBCs, but fewer mutations than GC B cells [68, 72]. However, T‐bet^+^ MBCs have overlapping gene signatures and the highest clonality overlap with GC B cells, suggesting they may have spent time in the GC or differentiated from a GC precursor. Despite low affinity maturation, ABCs are enriched in HIV envelope‐specific B cells, suggesting a high baseline specificity. ABC expansion correlates with viral load, supporting the notion that they are generated in response to strong initial BCR signaling [13, 69, 72]. Therefore, upregulation of BCR inhibitory molecules could reflect a response to dampen these strong BCR signals.

ABC hyporesponsiveness to BCR stimulation may reflect a persistent inflammatory environment rather than cellular exhaustion. As shown in several studies, stimulating ABCs in vitro with anti‐BCR does not result in proliferation, cytokine secretion, or differentiation to ASCs [5, 68, 73]. However, these systems promote BCR cross‐linking to soluble antigens, and when tested with membrane‐bound stimulation via lipid bilayers, ABCs robustly respond [74]. Membrane‐bound antigen led to the inhibitory receptor FcγR2b, which is upregulated on atypical B cells, being excluded from colocalizing with the BCR [68, 74]. Whether this is similar to other inhibitory markers is unknown, but it supports ABCs in chronic responses that require the acquisition of membrane‐bound antigen, likely from follicular dendritic cells (FDCs), to sustain effector responses. Unlike in other responses, TLR signaling does not seem to promote further differentiation, as atypical MBCs in LNs do not have a TLR signaling signature, nor do they respond to CpG stimulation in vitro [68, 72]. Additionally, transfer of atypical or classical MBCs from *Plasmodium* sporozoites immunization resulted in less differentiation to ASCs and GC B cells by donor atypical B cells after rechallenge [51], suggesting they may not expand or survive as well as classical MBCs. Peripheral tissue responses are not as well studied, but the increase in the migration markers CXCR3 and CCR6 supports their homing to inflamed tissue [13, 69, 72]. Therefore, ABCs in the context of chronic infections are not necessarily defective; they may require higher or alternative activation requirements, caused by the persistent inflammatory environment, to drive efficient effector responses.

### 3.3 Influenza

Studying T‐bet^+^ B cells in the context of influenza infection gives us insight into the unique functional properties of ABCs. Namely, as the acute infection resolves, the lymphoid microenvironment remains intact because the infection is restricted to the lungs. In addition, studies primarily utilize antigen tetramers to identify virus‐specific B cells. While valuable, this could lead to different interpretations of ABC characteristics, as tetramer‐positive cells will exclude lower‐affinity cells, as seen in other infection models, and prioritize the analysis of higher quality, probably longer‐lived, clones. Protocols to generate other antigen tetramers, like those for LCMV, are now established and can shed light on how low‐vs high‐affinity affect ABC characteristics [75]. Additionally, T‐bet expression in B cells appears to contribute to MBC formation during influenza infections. T‐bet is required for IgG2c antibody production in mice and IgG1 in humans, which preferentially bind to activating FcγRs, whose engagement regulates a protective immune response against many viral infections, especially influenza [76, 77]. The IgG2c isotype has been found to be enriched for antibodies that target the HA‐stalk regions, which could provide broad protection against heterotypic strains [27]. Further phenotyping to determine if these cells are ABCs or other T‐bet‐expressing populations is not always available. Thus, these studies likely demonstrate T‐bet attributes in mixed populations.

In contrast to the above infections, GC B cell development is well characterized from influenza infections and the extensive longevity of GCs is a known feature that produces high affinity MBCs. Many studies have suggested that the long‐lived antigen‐specific MBCs found in mediastinal lymph nodes (mLN) are primarily derived from the GC, whereas reports on cells from the spleen and lung have yielded contrasting findings regarding whether they are derived primarily from the GC or EF response [27, 29, 30, 78]. Nevertheless, MBCs seed the lungs early in infection and are known to establish resident memory responses that quickly differentiate into ASCs upon restimulation [79]. This seeding of MBCs in the lungs requires local antigen encounter, which may explain why early recruitment is critical; however, eventual downregulation of BCR signaling is needed to establish residence [80, 81]. These resident B cells do not recirculate, but they do provide protection against reinfection by mounting a rapid ASC response independent of circulating cells.

Following influenza infection, T‐bet^+^ B cells exhibit distinct tissue‐specific phenotypes and localization attributes (Figure 3B). Identification of these cells is typically characterized by CXCR3 expression or utilizing T‐bet‐reporter mice [27, 30, 43]. These antigen‐specific T‐bet^+^ cells can be found in the spleen, lung, and mLN, but the mLN lacked cells expressing the highest level of T‐bet [27]. These T‐bet^hi^ cells were shown to be splenic resident and largely excluded from circulation and lymphatics, but could be found in the lungs and spleen 100 days after primary infection [27, 43]. In contrast, MBCs with low or no T‐bet expression were able to recirculate. While VH sequencing suggests that T‐bet^+^ and T‐bet^−^ cells share common precursors, they do not interconvert, suggesting they have received distinct signals that confer their T‐bet expression and localization properties. To become lung resident, pre‐memory cells in the GCs of the mLN need IFN‐γ from Tfh cells to become CXCR3^+^ memory cells that migrate to the lungs [30]. GC B cells that have an IFN‐γ gene signature are specifically enriched for becoming lung resident cells. Not having the IFN‐γ‐STAT1‐T‐bet signaling pathway results in reduced lung resident memory B cells and also their earlier GC‐derived precursors. This was especially true for lung CD80^+^ T‐bet^+^ MBCs, which require this signature for ASC differentiation and maintenance in the lung [43]. While CXCR3 is a common receptor that cells use to home to inflamed tissues, it is not absolutely required [30]. However, once in the lungs, it could help guide cells to the inflamed areas, thereby aiding effective differentiation into ASCs following restimulation. As such, induced T‐bet‐deficient models demonstrate that depleting T‐bet after day 30 post infection leads to a defect in ASC differentiation following rechallenge [43]. Thus, long‐term T‐bet expression is important for maintaining lung resident B cells in a state that is optimal for differentiation.

While these studies demonstrate that T cell‐derived IFN‐γ is important for driving ABC‐like lung memory B cells, the extent to which TLR signaling (specifically TLR7 or TLR9) or IL‐21 signaling is required to promote ABCs during influenza infection remains unknown. Furthermore, because not all studies utilize CD11c or other ABC markers, it is unclear whether these findings can be attributed to this subset per se or whether the overall response to influenza infection requires T‐bet expression independent of the ABC subset. These studies highlight that antigen‐specificity and development in the GC of mLN seem to be the signals ABCs need to become long‐lived tissue resident cells that quickly differentiate into ASCs to provide a rapid protective response in subsequent infections. The functions of splenic resident or EF‐derived ABCs in long‐term protection against influenza infection have not yet been explored.

### 3.4 Vaccination in Humans

ABCs have been found to arise in the blood following vaccination. These studies used different markers to define their ABC‐like population, including CD21, FcRL5, CD11c, and CD27. One study found that CD21^lo^, CD11c^+^, and FcRL4^+^ expression were most useful in separating ABCs from MBCs in the tonsils of healthy donors [51]. SARS‐CoV‐2 vaccination utilized CD11c^+^ and FcRL5^+^ to classify its ABC‐like populations, whereas influenza vaccination used CD21^lo^, FcRL5^+^, and CD27^+^ to distinguish its ABC‐like populations [55, 82]. Nevertheless, high T‐bet expression was confirmed in these populations, although it was not limited to these ABC‐like cells. This potentially reflects transient T‐bet expression in subsets that differ from those with long‐term expression as seen in ABCs. Additionally, populations with slightly different expression profiles of CD21, CD85j, CD62L, and CD27 exhibit distinct kinetics: circulatory cells peak early and remain stable, whereas ABC‐like cells peak late and then quickly subside [83]. This suggests that blood sampling after vaccination would be enriched for different circulating populations depending on the timing relative to immunization. The lack of a specific population could represent a systemic decrease or homing to tissue sites for long‐term residency.

Isolating memory B cells from blood does not permit analysis of their developmental stimuli and origins. However, patients with deficiencies in IFN‐γ, STAT1, T‐BET, and ZEB2 all have reduced ABCs, suggesting similar developmental pathways. Upon boosting with the Pfizer BNT162b2 SARS‐CoV‐2 vaccine, T‐bet^+^ cells expand and progressively become isotype switched and mutated. Therefore, repeat vaccinations are needed to produce affinity mature clones. Despite these enhancements, ABCs had similar isotype profiles and mutation frequencies to those of switched memory or FcRL5^−^ B cell populations after SARS‐CoV‐2 and influenza vaccination, respectively [55, 82]. These similarities in affinity maturation obscure interpretations of whether they are GC or EF‐derived. T‐bet^hi^ cells are enriched for receptor binding domain (RBD)^+^ clones after SARS‐CoV‐2 vaccination compared with other B cell subsets. This suggests T‐bet^hi^ B cells have higher germline specificity as they are not undergoing extensive affinity maturation. This higher affinity, however, does not promote a poised ASC fate as transcripts or open chromatin were not enriched for ASC genes such as IRF4, PRDM1, or XBP1 following either SARS‐CoV‐2 or influenza vaccination [55, 82]. Regardless, they are enriched for gene sets, including antigen presentation, activation, and proliferation pathways, suggesting they are effector memory cells. Metabolic pathways that could sustain ASC function and differentiation, such as mTORC1, oxidative stress, and fatty acid metabolism, and genes found in protein production and catabolism, were enriched in their ABC‐like population expressing FcRL5 [82]. Cells lacking FcRL5 were enriched for central memory genes and stem‐like transcription factors TCF7 and LEF1. While these gene profiles certainly support the idea that ABC‐like cells are primed to become ASCs, they also suggest they are in a highly active state that would more likely produce any effector response, including ASC differentiation. When stimulated ex vivo to promote ASC formation, T‐bet^+^ B cells from immunized patients had reduced survival but produced more ASCs than other memory subsets [55, 82]. This seems to depend on antigen specificity, as, when normalized to antigen‐specific cells, they differentiated similarly to other switched MBC [55]. Taken together, these data suggest that vaccination can induce an effector memory T‐bet B cell population that is antigen‐specific and transcriptionally poised for effector responses. This enrichment for antigen specificity likely reflects stronger BCR signals, promoting a highly active state ideal for differentiation in response to further stimulation.

The more active state of ABCs following vaccination could come at the cost of survival, as after 180 days post SARS‐CoV‐2 booster vaccination, the T‐bet^+^ B cell frequency decreased in the blood RBD^+^ pool [55]. This was similar in another cohort, where the DN2 and ABC subsets accounted for a large proportion of the spike‐specific pool 1 week after vaccination but declined by the second week [84]. Alternatively, T‐bet^+^ cells could seed tissues or undergo phenotypic changes over time. As tissue inflammation during infection is not recapitulated during vaccination, it is unclear which tissues T‐bet^+^ cells would reside in following immunization, or whether they could promote a protective response within these sites. Aged individuals or those who do not mount an effective and durable response to influenza vaccines have more ABCs [85]. This correlation suggests that promoting ABCs through typical vaccination strategies may not confer protection. Because there is no tissue‐specific inflammation during vaccination, memory cells are likely to reside in the spleen or lymph nodes and respond to systemic antigens or inflammatory cues after infection. These non‐local responses may lead to a distant, delayed response that may not align with the protective functions of ABCs. Taken together, T‐bet^+^ B cells exhibit a heightened activation state after vaccination, which could allow for rapid antigen‐specific differentiation upon restimulation. This effector memory profile may confer better protection against reinfection, but may not provide long‐lasting protection or be localized at sites of infection. Therefore, more studies are needed to determine whether the T‐bet^+^ memory B cells measured in these studies leave circulation and enter tissues during human vaccination, thereby supporting long‐term survival and optimal effector potential.

## 4 Autoimmune Diseases

ABCs have been shown to have a pathogenic role in several autoimmune diseases, such as systemic lupus erythematosus (SLE, lupus) [8, 17, 49], rheumatoid arthritis (RA) [86, 87], Sjogren's disease (SjD) [88], and multiple sclerosis (MS) [89]. While the expansion of ABCs in patients with autoimmune diseases is most commonly studied in the circulation, tissue‐resident ABCs play an important and underappreciated role. ABCs are believed to generate autoantibodies locally in inflamed target organs, independent of systemic B cell responses in secondary lymphoid tissues. In these tissues, ABCs may play a role in disease development, via antigen presentation, autoantibody production, and cytokine secretion, and have been suggested to participate in secondary GCs [90, 91, 92, 93, 94]. The development of ABCs in these tissues is similar to that of secondary lymphoid organs, as developmental signals such as TLR7 and TLR9 and cytokines IL‐21 and IFN‐γ are typically elevated in these tissues during autoimmunity [17, 95]. There have been several studies in mice and humans assessing ABCs in tissues in the context of autoimmunity, yet the exact local and systemic contributions of these ABCs to these diseases remain unclear.

### 4.1 Systemic Lupus Erythematosus

SLE is a chronic autoimmune disease with a female bias and marked by increased activation of CD4^+^ T cells and B cells, which correlates with disease activity. A key characteristic of the disease is the presence of antinuclear autoantibodies, which lead to immune complex deposition and an inflammatory response, causing damage to organs such as the kidneys [96]. Accumulation of ABCs (typically referred to as DN2 B cells in these studies) has been observed in SLE patients and lupus mouse models, and these cells are prone to secrete abundant autoantibodies during the pathogenesis of systemic autoimmunity, as well as contributing to innate‐like autoreactive responses. The increase in ABC/DN2s is positively correlated with elevated serum levels of IFN‐I and anti‐chromatin IgG in SLE [97]. Depletion of CD11c B cells in mice using diphtheria toxin reduced anti‐chromatin IgG and disease manifestations [98]. Other studies in SLE patients found that CD11c^+^ DN2 B cells correlated with serum levels of anti‐SM and anti‐RNP IgG antibodies [17]. While ABCs were originally characterized in the peripheral blood of SLE patients, a growing body of evidence has established that these B cells accumulate in principal organs targeted in lupus, including the kidney, skin, and spleen [6, 99, 100, 101]. Understanding where ABCs reside within SLE tissues, how they adapt to organ‐specific microenvironments, and the distinct pathogenic functions they execute in each compartment are essential to developing more precise therapeutic strategies in SLE.

Much of our understanding of ABC biology in lupus has been established from studying these B cells in the spleen of lupus‐prone mice. The spleen is the main secondary lymphoid organ in which ABC development and expansion may be initiated in SLE [102, 103]. The frequency of CD11b and CD11c expressing ABCs is elevated in the spleens of several lupus‐prone models such as NZB/WF1 mice, MRL/Lpr, SLE1.Yaa, and pristane treated mice [90, 102, 104, 105]. Splenic ABCs in lupus‐prone mice are not a static population but undergo dynamic oligoclonal expansion and further differentiation. In a lupus‐like murine model driven by the absence of DEF6 and SWAP‐70, ABCs showed sex‐specific differences in cell number, upregulation of an interferon‐stimulated gene (ISG) signature, and oligoclonal expansion, differentiating into both CD11c^+^ and CD11c^−^ effector B cell populations with pathogenic and pro‐inflammatory functions [94]. The original assessment of ABCs in lupus‐prone NZB/WF1 mice used flow cytometry to reveal that splenic ABCs were positive for B220, IgM, CD5, CD11b, CD11c, CD19, and CD138, and expressed high levels of Fas, CD80, CD86, CD122, VCAM‐1, and MHC‐II [6]. The high‐level expression of MHC‐II and co‐stimulatory molecules on splenic ABCs identifies them as potent antigen‐presenting cells; however, CD138 expression suggests their differentiation into ASCs. Their positioning at the T cell/B cell border within the spleen allows them to efficiently activate autoreactive T cells and establish the self‐amplifying pathogenic loops that sustain systemic autoimmunity. This interaction may promote subsequent ASC differentiation. In chronic graft‐versus‐host disease‐induced lupus mice, elevated serum levels of anti‐chromatin IgG2a and increased ABCs were observed, with high CD138 expression indicating ABCs were at a pre‐plasma cell differentiation stage, secreting abundant anti‐chromatin IgG2a. Depletion of CD11c^+^ B cells or T‐bet deficiency in B cells alleviated these autoantibody levels [103]. Therefore, the spleen is a developmental niche for ABCs during SLE in several models, amplifying pathology by promoting T cell activation and ASC differentiation during disease progression.

The kidney is a critical target organ for immune complex deposition in SLE, and intrarenal ABC accumulation has emerged as a key feature of lupus nephritis [101]. Single‐cell RNA sequencing of kidney biopsies from patients with lupus nephritis (LuN) revealed that the intrarenal immune landscape is profoundly shaped by ABC biology (Figure 3C). In active disease, activated B cells showed a gene profile consistent with an ABC signature and an IFN‐I response, observed in most renal immune cell populations [101]. These findings suggest that ABCs are not merely trafficking through the kidney but are locally activated within the organ, responding to the IFN‐I milieu found in LuN. An IFN‐I signature was also observed in lupus‐prone mice, with the female‐biased ABC expansion being overridden by TLR7 duplication in males [94]. Clonal comparisons of intrarenal ABCs have shed light on their functional identities and clonal relationships. Paired single‐cell sequencing of B and T cells from the kidneys and peripheral blood of patients with active LuN identified high infiltration of intrarenal ABCs and ASCs, which were the dominant B cell populations in the kidney compartment. Additionally, a distinct expansion of IGHG4‐59 isotype, specifically enriched in the kidney, showed clonal overlap between ABCs and ASCs [106]. This direct clonal overlap between intrarenal ABCs and ASCs suggests that kidney ABCs are functional precursors to local antibody‐secreting cells, contributing directly to the immune complex deposition that drives glomerulonephritis. Spatial transcriptomics of childhood‐onset LuN kidneys demonstrated that B cells clustering within the tubulointerstitium harbor and sustain ABC‐enriched immune aggregates [107]. ABCs may be tissue‐resident cells located in specific renal anatomical niches, as the kidneys of LuN patients have been shown to have elevated sodium concentrations that promote B cell survival. The intrarenal ABCs may serve as an in situ reservoir for autoantibody production, directly fueling immune complex deposition, which contributes to glomerular injury and tubulointerstitial inflammation. Kidneys in LuN have also been shown to exhibit low oxygen tension and hypertonicity, with infiltrating T cells and macrophages upregulating HIF‐1α in response to hypoxia [108]. This environmental stress in the kidney may help shape the development and function of internal B cells [109]. For instance, HIF‐1α driven metabolic programming may alter transcription and promote ABCs to become more inflammatory and to secrete cytokines. However, other studies have found that intrarenal GZMK^+^IFNG^+^ CD8^+^ T cells and ABCs co‐localize within tertiary lymphoid structures (TLS) in the kidney, suggesting that GZMK^+^ CD8 T cells drive local ABC differentiation through IFN‐γ [106]. Therefore, the inflammatory and metabolic environment of the autoimmune kidney promotes ABC pathology.

Cutaneous involvement is among the most prevalent manifestations of SLE, and ABCs have emerged as key drivers of the B cell‐mediated pathology within lupus lesions. In a humanized cutaneous mouse model, IL‐21 and TLR7/9 signaling were shown to recruit ABCs into cutaneous lupus lesions, where they locally produced IgG autoantibodies [100]. In human disease, the local skin microenvironment is also characterized by elevated IFN‐I production in response to nucleic acid‐containing immune complexes and ultraviolet‐triggered keratinocyte apoptosis. This would provide a suitable environment for providing local TLR7/TLR9 activation of ABCs in the skin. There is a significant increase in B cell signatures in lesional skin from patients with isolated cutaneous lupus compared with similar lesions from patients with systemic lupus [110]. The accumulation of ABC‐like B cells in these lesions tends to be in the most fibrotic and treatment‐resistant patients. This suggests that in the skin of these patients, ABCs may contribute to the chronicity and scarring characteristic of the lesions and that skin‐resident ABCs may act as an independent driver of local pathology [111]. However, in cutaneous lupus, it is unknown whether ABCs arise locally in the skin or whether the expanded circulating ABCs in SLE may migrate and contribute to the expansion in the lesions.

### 4.2 Rheumatoid Arthritis

RA is a chronic systemic autoimmune disease characterized by immune cell infiltration into the joint lining [112]. The expansion of activated immune cells in the synovium leads to cartilage destruction and the generation of autoantibodies, such as rheumatoid factor and anti‐citrullinated protein antibodies [113]. ABCs are among the most elevated B cell populations in the blood, synovial fluid, and synovial tissue of patients with RA and have been shown to be positively correlated with disease activity (Figure 3D) [114]. Single‐cell studies have revealed distinct transcriptomic features between B cells in the blood and those in the synovium, suggesting a role for the tissue microenvironment in the pathogenesis of ABCs at these sites [114].

The phenotype of circulating ABCs in RA blood is similar to that in SLE, with elevated T‐bet and CD11c, but lacks CD27 and CD21. Moreover, circulating ABCs showed high expression of IL‐13Rα1 and the IL‐4 receptor but lacked the plasma cell marker BCMA [115]. Although IL‐4 has been shown to downregulate ABC formation in SLE, ABCs from RA patients produce more IL‐4 than CD11c^−^ B cells [115]. IL‐13Rα1 on ABCs has also been reported to promote their development in RA and lupus mice [116]. This suggests potential differences in the developmental pathways of ABCs in disease. In SLE, circulating ABCs stimulated with TLR7 or TLR9 can promote IL‐4 and IL‐10 secretion, but in RA, they produced IL‐12, IL‐17, IL‐23, and low amounts of IL‐10 [5, 86, 114]. Circulating ABCs in RA expressed CCR2 and CXCR3, whose ligands, CCL2 and CXCL9,10, and 11, respectively, are elevated in the synovium [86, 117, 118]. Receptor expression may promote the migration of circulating ABCs into the synovium in RA, thereby promoting disease.

ABCs in the synovium of RA patients can promote disease by producing autoantibodies, secreting proinflammatory cytokines, and presenting antigens. While circulating ABCs may migrate into the synovium, those present in the tissue showed elevated expression of activation markers, including HLA‐DR, CD40, CD69, and Ki‐67. This suggests active local differentiation into antibody‐secreting cells rather than mere trafficking and differentiation [86]. ABCs isolated from synovium differentiated into ASCs ex vivo to produce rheumatoid factor and anticitrullinated protein autoantibodies [119]. ABCs in synovial fluid express higher proportions of IgG and IgA compared to their peripheral blood counterparts. ABCs from RA patients have been shown to activate fibroblast‐like synoviocytes (FLSs) by releasing TNF‐α, driving synovial inflammation through ERK1/2 and JAK‐STAT1 signaling pathways, and promoting the secretion of IL‐6, MMP‐1, MMP‐3, and MMP‐13. The ABC‐FLS facilitated proinflammatory interaction was dependent on CXCL12 and VCAM‐1 [120]. Thus, in the synovium, ABCs and FLS form a pathogenic loop in which ABCs and synovial fibroblasts mutually sustain each other's activation. ABCs in the synovium have also been shown to utilize their antigen‐presenting capacity to activate T cells, thereby driving disease. In studies of early RA patients, ABCs express high levels of HLA‐DR and exhibit a secretion profile that includes IL‐12p70 and IL‐23, but low levels of the regulatory cytokine IL‐1 [86]. Taken together, this suggests that while autoreactive ABCs may migrate from the blood to the synovium, there may also be a local population with discrete functions that promote disease in RA.

### 4.3 Sjögren's Disease

SjD is a chronic systemic autoimmune disease primarily targeting the exocrine glands, most notably the salivary and lacrimal glands [121]. B cells have been shown to become dysregulated in the exocrine glands of SjD patients, with B cell lymphoma development being 15–20 times more likely than in individuals without SjD. B cells accumulate within the salivary glands alongside CD4+ T cells, forming periductal lymphocytic foci [122]. B cells in this focus have been shown to produce pathogenic anti‐Ro/SSA and anti‐La/SSB antibodies, which are present in the majority of SjD patients [88]. ABCs have been recently identified as a pathogenic contributor specifically enriched in SjD salivary glands. A study of 44 SjD patients showed that ABCs infiltrate the labial salivary glands, driven by IL‐21 from CD4^+^ T cells and autocrine IFN‐γ [88]. In murine models of SjD, ABC accumulation in salivary glands preceded the formation of lymphocytic foci and correlated with Tfh cell expansion, while TLR7 signaling was identified as a key driver of ABC expansion in a MyD88‐dependent manner [123, 124]. While B cells have been shown to produce IL‐6, TNF, and lymphotoxin and to have APC functions in these glands, ABCs are thought to primarily produce autoantibodies [92, 125]. Collectively, these findings position ABCs as pathogenic contributors to SjD‐associated salivary gland inflammation and potential targets for future therapeutic intervention.

### 4.4 Multiple Sclerosis

MS is a chronic inflammatory demyelinating disease of the central nervous system (CNS) affecting over 2.5 million people worldwide [126]. A hallmark of MS is the presence of oligoclonal bands in the cerebrospinal fluid (CSF), reflecting clonal expansion and intrathecal immunoglobulin synthesis by B cells and plasma cells within the CNS compartment [127]. B cell depletion therapy is an effective treatment for MS, but inadequate depletion of some B cells in the CNS parenchyma may lead to more progressive disease [128]. ABCs have emerged as a relevant B cell subset in MS pathogenesis, though their precise role remains less fully characterized than in other autoimmune diseases such as SLE. ABCs were first phenotypically identified by CyTOF in the peripheral blood of early MS patients during their first neurological relapse [89]. scRNA‐seq and flow cytometry analysis of early MS PBMCs compared to healthy controls demonstrated that CD19^+^CD20^+^CD21^lo^CD11c^+^T‐bet^+^ ABCs were significantly enriched in MS. Moreover, this study showed that these ABCs upregulated inflammatory cytokine transcripts, including CXCL8, IL‐18, VEGFA, and CXCR3 [129]. ABCs have been examined across models of EBV infection, murine gammaherpesvirus 68 (γHV68), MS, and experimental autoimmune encephalomyelitis (EAE, a mouse model of MS), demonstrating that viral infection and autoimmunity drive the functional ABC population required to promote γHV68‐enhanced EAE disease [130]. Functionally, ABCs in MS, as in other autoimmune diseases, are capable of producing proinflammatory cytokines, including IFN‐γ and TNF‐α, presenting antigen to T cells, and contributing to humoral autoimmune responses [131, 132, 133]. Taken together, the current body of evidence positions ABCs as expanded, inflammatory, and disease activity‐correlated in MS, with a potentially unique role as a cellular nexus between prior EBV infection and CNS‐directed autoimmunity. However, key questions, including whether ABCs traffic to the intrathecal compartment, whether they are clonally expanded within the CSF, and whether they represent a therapeutically distinct target from bulk B cell depletion, remain unanswered.

## 5 Cancer

The tumor microenvironment (TME) is a complex milieu in which immune cells, stromal cells, and malignant cells interact to shape tumor progression and treatment outcomes. While B cells have not been as well studied as T cells in the TME, they have been shown to infiltrate tumors and modulate tumor control and immune evasion [134, 135]. ABCs are among the B cell populations shown to accumulate in tumor tissue [136]. In certain tumors, such as diffuse large B‐cell lymphoma (DLBCL), CD11c^+^T‐bet^+^ B cells appeared to be expanded in the latter stages of disease [137]. There is evidence that ABCs are a functional B cell population in the TME. As in the tissue microenvironment of autoimmunity, ABCs in the TME contribute to local immunity through antigen presentation and cytokine secretion, but also suppress immune responses via IL‐10. In lung adenocarcinoma (LUAD), the presence of CD11c^+^ B cells with an effector memory phenotype has been correlated with improved overall patient survival in treatment‐naive cohorts [138]. Thus, there is growing evidence that tumor‐infiltrating B cells are critical modulators of anti‐tumor immunity, influencing both tumor control and immune evasion.

Central to B cell activity in the TME is their organization into TLS. TLS are ectopic lymphoid aggregates that develop within non‐lymphoid tissues under conditions of chronic inflammation, including tumors [135]. TLS serve as local sites of antigen presentation, lymphocyte activation, and humoral immune responses [139]. Their architecture recapitulates features of secondary lymphoid organs, including organized T and B cell zones, FDC networks, high endothelial venules, and an environment that supports active B cell somatic hypermutation and affinity maturation [139, 140]. The presence of tumor‐associated TLS has been associated with improved overall survival in several cancers, such as melanoma, renal cell carcinoma (RCC), colorectal cancer, bladder cancer, and pancreatic ductal adenocarcinoma [139, 141, 142]. In tumors without mature TLS, B cells are either scarce or, if present, have a more regulatory, immunosuppressive phenotype. In tumors with TLS, B cells are activated, undergo antibody class switching and somatic hypermutation, and subsequently differentiate into plasma cells that produce IgG or IgA antibodies targeting tumor‐associated antigens [135]. Specifically, spatial transcriptomic characterization of mature TLS in head and neck squamous cell carcinoma (HNSCC) revealed enrichment of CD4^+^ T cells that support B cell activity, whereas non‐mature TLS tumors fail to support these cells [143]. In some tumor‐associated TLS, B cells also secrete cytokines including TNF, IL‐2, IL‐6, and IFN‐γ, which recruit T cells and other effector cells to drive cytotoxicity and phagocytosis [135].

While there is evidence of ABC in tumors, many studies have found that the majority of B cell activity in the TME is localized to discrete regions of TLS. ABCs have been shown to promote TLS formation in cancer. In a murine hepatocellular carcinoma (HCC) model, it was shown that ABCs were increased and expanded in liver TLS with cancer progression. Blocking T‐bet expression in these B cells resulted in smaller TLS and reduced tumor lesion size, suggesting a role for ABCs in TLS formation and growth [144]. However, a study of B cells from TLS in HCC patients revealed a more regulatory phenotype. Single‐cell studies of B cells from the TLS identified a population with an ABC phenotype that upregulated PD‐L1, CD25, and granzyme B expression [145]. Moreover, trajectory analysis suggested that TLS environments transition these ABCs toward a more immunosuppressive cell state, characterized by FCRL4 and IL‐10 expression [145]. A pan‐cancer scRNA‐seq atlas identified key subset signatures, including atypical memory B cells (expressing ITGAX and FCRL5) with an exhausted phenotype and progenitor potential to differentiate into antibody‐secreting cells [146]. This atlas identified subset‐specific effects on overall immune checkpoint blockade response, confirming that the atypical/ABC subset is functionally distinct from GC B cells and differentially influences immunotherapy outcomes. Spatially validated ligand‐receptor pair analyses further revealed unique crosstalk between atypical B cells and CD4^+^ T cells, suggesting non‐canonical interactions with implications for TLS organization [146]. Thus, ABCs represent a biologically complex and functionally heterogeneous B cell population whose roles in tumor‐associated TLS are context‐dependent and incompletely understood. Evidence from HCC, NSCLC, HNSCC, and melanoma collectively reveals that ABCs can both promote TLS architectural formation and, paradoxically, acquire immunosuppressive Breg phenotypes within those structures (Figure 3E). Their dual capacity to contribute to local immunity via antigen presentation and cytokine secretion on one hand, and to suppress immune responses via IL‐10 and regulatory Breg programs on the other makes them a compelling but paradoxical subject of investigation in the TLS of tumors.

## 6 Conclusion

In summary, ABCs are a versatile subset of B cells whose biology is influenced by the tissue environments where they develop and function. The generation of ABCs depends on key signals, including TLR7/9 signaling, IFN‐γ, IL‐21, and assistance from effector CD4 T cells. However, it is the local tissue microenvironment that ultimately shapes the phenotype, localization, and effector functions of ABCs. In various contexts such as infections, autoimmune diseases, and cancer, specific tissue environments create distinct transcriptional, metabolic, and cellular signals that influence whether ABCs function as protective agents, harmful drivers, or immunosuppressive regulators. The phenotypic heterogeneity that has long complicated the classification of ABCs likely reflects this environmental plasticity rather than representing distinct lineages. As single‐cell and spatial transcriptomic technologies continue to advance, a more precise mapping of ABC states within defined tissue compartments will be essential for resolving these outstanding questions. Furthermore, utilizing longitudinal sampling of tissue‐resident ABCs across disease states and developing humanized models should help clarify how organ‐specific microenvironments influence these B cells. Identifying the signals that toggle ABCs between protective and pathogenic states could open new therapeutic avenues. Vaccine strategies against pathogens could focus on enhancing long‐lasting tissue‐resident ABC responses. In cases of autoimmunity and cancer, targeted interventions may selectively disrupt ABC‐driven pathology in the affected organs or the tumor microenvironment. Ultimately, viewing ABCs not as a fixed population but as a microenvironmentally instructed continuum will be crucial for unlocking their full biological and therapeutic significance.

## Funding

This work was supported by the National Institutes of Health (R01AR073912, R21AI190857).

## Conflicts of Interest

The authors declare no conflicts of interest.

## Data Availability

The authors have nothing to report.
